# Expanding and validating the biomarkers for mitochondrial diseases

**DOI:** 10.1007/s00109-020-01967-y

**Published:** 2020-08-26

**Authors:** Alessandra Maresca, Valentina Del Dotto, Martina Romagnoli, Chiara La Morgia, Lidia Di Vito, Mariantonietta Capristo, Maria Lucia Valentino, Valerio Carelli, the ER-MITO Study Group

**Affiliations:** 1 IRCCS Istituto delle Scienze Neurologiche di Bologna, UOC Clinica Neurologica, Bologna, Italy; 2 Department of Biomedical and Neuromotor Sciences, University of Bologna, Bologna, Italy

**Keywords:** Biomarkers, Mitochondrial diseases, Cell free circulating-mtDNA, Creatine, FGF21, GDF-15

## Abstract

**Abstract:**

Mitochondrial diseases are highly heterogeneous metabolic disorders caused by genetic alterations in the mitochondrial DNA (mtDNA) or in the nuclear genome. In this study, we investigated a panel of blood biomarkers in a cohort of 123 mitochondrial patients, with prominent neurological and muscular manifestations. These biomarkers included creatine, fibroblast growth factor 21 (FGF21) and growth/differentiation factor 15 (GDF-15), and the novel cell free circulating-mtDNA (ccf-mtDNA). All biomarkers were significantly increased in the patient group. After stratification by the specific phenotypes, ccf-mtDNA was significantly increased in the Mitochondrial Encephalomyopathy Lactic Acidosis Stroke-like episodes syndrome (MELAS) group, and FGF21 and GDF-15 were significantly elevated in patients with MELAS and Myoclonic Epilepsy Ragged Red Fibers syndrome. On the contrary, in our cohort, creatine was not associated to a specific clinical phenotype. Longitudinal assessment in four MELAS patients showed increased levels of ccf-mtDNA in relation to acute events (stroke-like episodes/status epilepticus) or progression of neurodegeneration. Our results confirm the association of FGF21 and GDF-15 with mitochondrial translation defects due to tRNA mutations. Most notably, the novel ccf-mtDNA was strongly associated with MELAS and may be used for monitoring the disease course or to evaluate the efficacy of therapies, especially in the acute phase.

**Key messages:**

*• FGF21/GDF15 efficiently identifies mitochondrial diseases due to mutations in tRNA genes.*

*• The novel ccf-mtDNA is associated with MELAS and increases during acute events.*

*• Creatine only discriminates severe mitochondrial patients.*

*• FGF21, GDF-15, and ccf-mtDNA are possibly useful for monitoring therapy efficacy.*

**Electronic supplementary material:**

The online version of this article (10.1007/s00109-020-01967-y) contains supplementary material, which is available to authorized users.

## Introduction

Mitochondrial medicine, the field investigating mitochondrial diseases, just crossed 30 years of its existence [1]. Mitochondrial diseases are genetically determined metabolic disorders due to defective oxidative phosphorylation (OXPHOS), which may be caused by mutations affecting genes either encoded by mitochondrial DNA (mtDNA) or nuclear DNA (nDNA) [2]. When due to mtDNA mutations, mitochondrial diseases are in most cases maternally inherited or sometimes sporadic as for single mtDNA macrodeletions, obeying the peculiar rules of mitochondrial genetics. These include heteroplasmy/homoplasmy of the mutation, its mitotic segregation in somatic tissues, and the threshold effect, which are all relevant factors for the very variable clinical expression [3]. Conversely, when due to nDNA defects, mitochondrial diseases may follow any of the Mendelian inheritance patterns.

Mitochondrial diseases affect primarily organs and tissues with a high-energy requirement, usually causing encephalomyopathies [4]. However, they are frequently multi-systemic with a constellation of symptoms and signs, which may include cardiomyopathy and heart conduction defects, liver or kidney dysfunctions, diabetes mellitus, sensorineural deafness, isolated myopathy with ophthalmoplegia and ptosis, peripheral neuropathy, central nervous system manifestations such as stroke-like episodes (SLEs), epilepsy, ataxia and cognitive dysfunction, gastrointestinal dysmotility, retinal pathology, and optic nerve atrophy [2, 4]. They usually lead to major disability and, frequently, to premature death but may also present as a relentless, slowly progressive chronic condition or as an acute, life-threatening event needing an emergency treatment.

Because of the extreme complexity of OXPHOS functioning and regulation and its peculiar dual genetic dependence, the number of genes involved in mitochondrial disorders is very large, potentially coinciding with the size of the mitochondrial proteome, which is thought to be composed by over 1500 proteins in humans [5]. Thus, even if individually rare, when taken as a whole, mitochondrial diseases are among the most frequent genetic diseases in humans. Just considering the minimum population prevalence of mitochondrial diseases due to mtDNA mutations, epidemiological studies provided estimates of ~ 1 in 10,000 individuals clinically affected at any age [6]. Even more impressively, pathogenic mtDNA mutations are found in approximately 1 in 200 of the background population [7], highlighting the massive reservoir of pathogenic mtDNA mutations in the general population. The minimum birth prevalence of mitochondrial diseases in children has been estimated of 13.1/100,000, again remarking that they are more common than lysosomal storage diseases and many other well-known neurogenetic diseases [8].

There is a very limited repertoire of effective treatments for mitochondrial diseases, and current clinical management is focused on treating complications and specific manifestations of the disease [9]. However, emerging therapeutic options are now available, as in the case of Leber’s Hereditary Optic Neuropathy (LHON) for which idebenone has been recently approved [10], and proof of principle for efficacy and feasibility of many other therapeutic strategies is provided [11], implicating the rapid increase of translation of these approaches into clinical trials [12].

Collaterally and instrumental to this scenario, an actively developing field in mitochondrial medicine is the identification of useful serum/plasma specific and sensitive biomarkers for diagnosis and follow-up of mitochondrial disease patients, as well as for evaluating the disease natural history and the response to therapy [13, 14]. Besides the traditional marker of OXPHOS dysfunction, the pathological increase of lactic acid [15], promising biomarkers for mitochondrial disease have been recently proposed, including fibroblast growth factor 21 (FGF21) [16, 17], GDF-15 [18], and creatine [19]. Similarly, also mitochondrial damage-associated molecular patterns (DAMPs), which are released after cellular disruption [20], may be useful for mitochondrial disease follow-up, but they have not been tested yet. In particular, the novel cell free circulating-mtDNA (ccf-mtDNA) biomarker [21] may be relevant for those disorders with acute events, possibly characterized by consistent loss of cellular integrity.

In our study, by taking advantage of an epidemiological survey of the Emilia-Romagna region in Italy (Emilia-Romagna-Mitochondria, ER-MITO), we investigated a panel of biomarkers in a large cohort of patients with mitochondrial diseases, consolidating results from previous reports and providing novel observations.

## Patients and methods

### Patients

We collected plasma and serum from 35 healthy individuals (mean age 41, standard deviation (SD) 11; males 15, females 20) and 123 patients (mean age 44, SD 16; males 76; females 47) affected by a mitochondrial disease caused by either mtDNA or nDNA genetic defects, largely derived from the epidemiological study Emilia-Romagna-Mitochondria (ER-MITO). The institutional ethical board (Comitato Etico Interaziendale Bologna-Imola) approved the study (CE-BI 13036), and all participants gave informed written consent. All procedures were performed according to the Declaration of Helsinki.

The cohort of mitochondrial patients is described in the Supplementary table 1 and included the following: Mitochondrial Encephalopathy, Lactic acidosis, and Stroke-like Episodes (MELAS) patients (*n* = 28), Myoclonic Epilepsy with Ragged Red Fibers (MERRF) patients (*n* = 6), Leber’s Hereditary Optic Neuropathy (LHON) patients (*n* = 34), Neuropathy, Ataxia, and Retinitis Pigmentosa (NARP) patients (*n* = 6), Optic Atrophy (OA) patients (*n* = 16), Chronic Progressive External Ophthalmoplegia (CPEO) patients (*n* = 17), Sensory Ataxia Neuropathy Dysarthria and Ophthalmoplegia (SANDO) patients (*n* = 4), and other mitochondrial encephalomyopathies (ME) (*n* = 12). For a few MELAS patients (*n* = 4), we had available longitudinal sampling over 3/4 years closely temporally related to SLEs and in the inter-critical periods.

The differences in number of samples analyzed for the four biomarkers depend on the number of aliquots available for each patient and control subject.

### Plasma and serum collection

Peripheral blood was collected in EDTA for plasma separation or in Serum Separation Tubes and centrifuged within 1 h from the collection. Whole blood was centrifuged 15 min at 2900 ×*g* for plasma and serum separation. Plasma for ccf-mtDNA analysis was further centrifuged for 10 min at 15000 ×*g* to avoid cell contamination. Aliquots were stored at − 80 °C until processing.

### Circulating cell free mitochondrial DNA assessment

Circulating cell free DNA (ccf-DNA) was extracted from 200 μl of plasma samples using the NucleoSpin® Plasma kit (Machery & Nagel), following manufacturer’s instructions. The circulating cell free-mtDNA (ccf-mtDNA) and cell free-nuclear DNA (ccf-nDNA) were quantified by droplet digital-PCR (dd-PCR, Bio-Rad) with Taqman-based methods, amplifying *MT-ND2* and *FASLG* [22] and *MT-ND1* [23]. The ccf-mtDNA and ccf-nDNA were expressed as copies of target gene on μl of template analyzed (copies/μl template).

### Heteroplasmy assessment of m.3243 and m.8344 mutations

DNA was extracted from white blood cells using the Maxwell® instrument (Promega). Heteroplasmy of m.3243A > G/*MT-TL1* and m.8344A > G/*MT-TK* mutations was quantified by dd-PCR using PrimePCR Custom Assays (Bio-Rad), discriminating the wild-type genomes from the mutated. Heteroplasmy was expressed as percentage of mutated mtDNA genomes on total mtDNA. Since the amount of m.3243 mutation in blood decreases with age, a correction was applied using the tool developed by the New Castle University (https://newcastlemitoapps.shinyapps.io/m3243ag_heteroplasmy_tool/) [24].

### Creatine assessment

For creatine evaluation, we excluded patients who were receiving creatine supplementation at the time of sampling. After serum filtration with 10 K molecular weight filter columns (Sigma-Aldrich), creatine was measured using a colorimetric assay (BioVision), following manufacturer’s instructions.

### FGF21 assessment

FGF21 was analyzed in serum samples using the Human Fibroblasts Growth Factor 21 AlphaLisa assay (PerkinElmer), following manufacturer’s instructions.

### GDF-15 assessment

GDF-15 was analyzed in serum samples using GDF15 Human ELISA Kit (Thermo Fischer), following manufacturer’s instructions.

### Statistical analysis

Statistical analyses were performed using GraphPad Prism 6.0 software and the statistical packages SPSS, version 20.0. Comparisons of control and MD groups were assessed by unpaired *t* tests with Welch’s correction. Differences between controls and the mitochondrial disease groups were analyzed using one-way ANOVA and Dunnett’s multiple comparisons tests. Univariate linear regression analysis was performed to assess the effect of age (independent variable) on each biomarker (dependent variable), after having checked for linearity between variables, homoscedasticity, and normality of the residuals.

Sensitivity and specificity ± 95% confidence interval (CI) were calculated after having set a threshold corresponding to the 95th percentile of control values [17]. The receiver-operating characteristic (ROC) curve and the area under the curve (AUC) were obtained using GraphPad Prism 6.0. For FGF21, three outliers identified in the control group by ROUT method (*Q* = 1%) were eliminated for the sensitivity/specificity, ROC curve, and AUC calculation.

Univariate linear regression models with Pearson’s correlations were used to test the possible associations between heteroplasmy and ccf-mtDNA in MELAS and MERRF patients and among the different biomarkers. For linear regression analysis, we considered value greater than ± 3SD as common cut-off criteria to identify residuals representative of outliers. Differences were considered statistically significant at *p* ≤ 0.05, and for Pearson’s correlation analyses, Bonferroni’s and Benjamini–Hochberg’s (FDR 0.10) correction methods were considered.

## Results

### Circulating cell free-mtDNA in plasma hallmarks MELAS

**Fig. 1:**
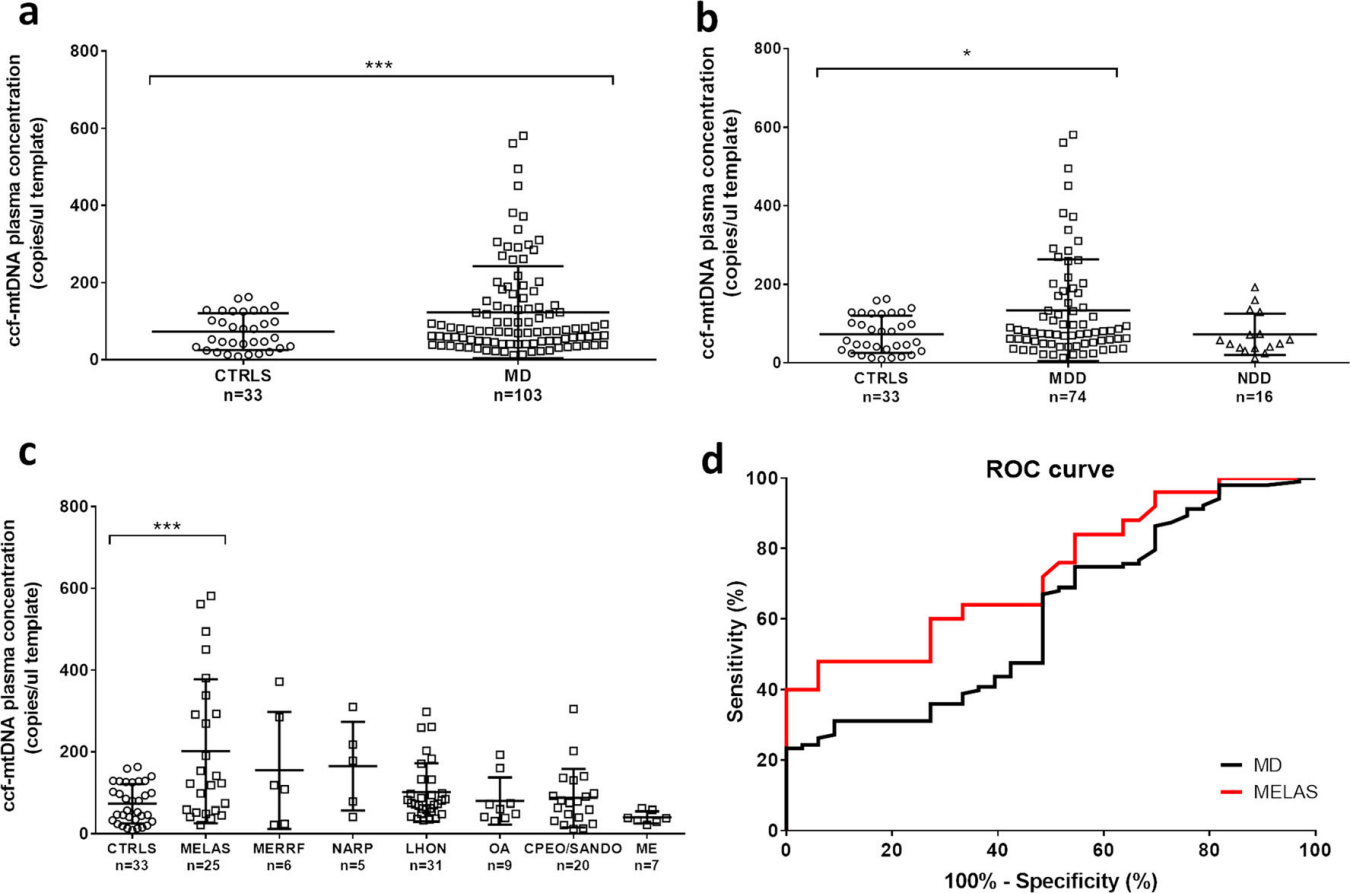
Evaluation of ccf-mtDNA in plasma from controls and mitochondrial patients. **a** Ccf-mtDNA (*MT-ND2*) in controls (CTRLS) and mitochondrial patients (MD). **b** Ccf-mtDNA (*MT-ND2*) in CTRLS, patients with mtDNA genetic defects (MDD), and nuclear DNA genetic defects (NDD). **c** Ccf-mtDNA (*MT-ND2*) in CTRLS and mitochondrial patients stratified by phenotypes. **d** ROC curves for ccf-mtDNA (*MT-ND2*) in MD and MELAS patients. AUC in MD was 0.61 (95% CI 0.50–0.72, *p* = 0.05), in MELAS 0.73 (95% CI 0.60–0.86, *p* < 0.01). *p value < 0.05, ***p value < 0.001

Mitochondrial DAMPs include ccf-mtDNA, which is currently a very active field of investigation in multiple diseases, including neurodegenerative disorders [21]. Thus, we valued this as a new possible biomarker particularly suited for mitochondrial diseases. Ccf-mtDNA was evaluated in plasma samples, amplifying two different regions of mtDNA (*MT-ND2* shown in Fig. 1 and *MT-ND1* shown in Supplementary Fig. 1). In parallel, also ccf-nDNA was assessed and shown in Supplementary Fig. 1.

We first compared our control group with the whole set of mitochondrial disease patients (MD), showing that ccf-mtDNA was significantly increased in MD patients (Table 1; Fig. 1a and Supplementary Fig. 1a). Separating the MD group into mtDNA genetic defects (MDD) and nuclear DNA defects (NDD), we found that ccf-mtDNA levels were significantly elevated exclusively in the MDD group (Fig. 1b and Supplementary Fig. 1b). Next, after further stratification by the specific mitochondrial phenotype, only the group of MELAS patients had significantly higher ccf-mtDNA, as compared with controls (Fig. 1c and Supplementary Fig. 1c). MERRF and NARP patients followed a similar tendency as MELAS, whereas for the LHON and CPEO/SANDO patients, only a few patients had increased ccf-mtDNA exceeding the highest values of the control range (Fig. 1c and Supplementary Fig. 1c). Comparable results were obtained with the two independent assays used for ccf-mtDNA, and correlation between the two data set is shown in Supplementary Fig. 1d. Assessment of ccf-nDNA showed no differences between controls and MD patients (Supplementary Fig. 1e-f).

**Table 1:** Descriptive analysis for all biomarkers in controls and mitochondrial patients

Biomarker		Mean	95% CI	Median	Standard deviation	Min	Max
Ccf-mtDNA MT-ND2 (copies/ul)	CTRLS	72.94	56.01–89.87	52.27	47.74	8.97	163.43
	MD	123.00	99.75–146.24	75.17	118.93	11.85	580.55
Creatine (umol/l)	CTRLS	6.92	4.79–9.06	6.85	5.61	0.00	19.07
	MD	12.11	9.52–14.69	11.16	10.83	0.00	50.91
FGF21 (pg/ml)	CTRLS	990.61	630.91–1350.31	659.39	909.29	88.52	3348.35
	MD	1765.76	1438.07–2093.44	1416.10	1374.26	0.00	7199.68
GDF-15 (pg/ml)	CTRLS	459.39	385.09–523.70	404.30	164.13	259.52	913.29
	MD	3712.74	2757.78–4667.79	2238.64	4004.98	318.65	25867.10

*CTRLS* controls, *MD* mitochondrial disease patients, *CI* confidence interval, *Min* minimum value, *Max* maximum value

Ccf-mtDNA increase in MD patients was not influenced by gender (Supplementary Fig. 2a-b) and age (Supplementary Fig. 2c-d).

Ccf-mtDNA (*MT-ND2* assay) sensitivity and specificity for MD patients were 25% (95% CI 17–34%) and 94% (95% CI 86–102%), respectively. Since the only significant difference was with MELAS group, we calculated ccf-mtDNA sensitivity for MELAS patients, which was 44% (95% CI 23–63%). We considered a cut-off threshold of 147 copies/ul, which represented the 95th percentile of control values. To verify the diagnostic power of ccf-mtDNA as biomarker for mitochondrial diseases and, more specifically, for MELAS patients, we generated the ROC curve for both groups (Fig. 1d). Although ccf-mtDNA had a weak capability in detecting MD patients, as indicated by the AUC of 0.61 (95% CI 0.50–0.72, *p* = 0.05), this biomarker was relatively efficient in the detection of MELAS patients, as indicated by an AUC of 0.73 (95% CI 0.60–0.86, *p* < 0.01).

Focusing on MELAS and MERRF as homogeneous group of mitochondrial disorders dependent on the heteroplasmic load for phenotypic expression, we correlated the ccf-mtDNA amounts with the corresponding heteroplasmy assessed in circulating blood cells. The heteroplasmy of the m.3243/MELAS mutation was normalized for the patient’s age, as recently suggested by Grady et al. [24], whereas this correction has not been applied to m.8344/MERRF mutation, for which there is no reliable information on the possible age-dependent rate of counter-selection against mutant mtDNA in blood cells. Overall, we failed to find any correlation between merged MELAS/MERRF heteroplasmy levels and ccf-mtDNA (Supplementary Fig. 2e-f).

**Fig. 2:**
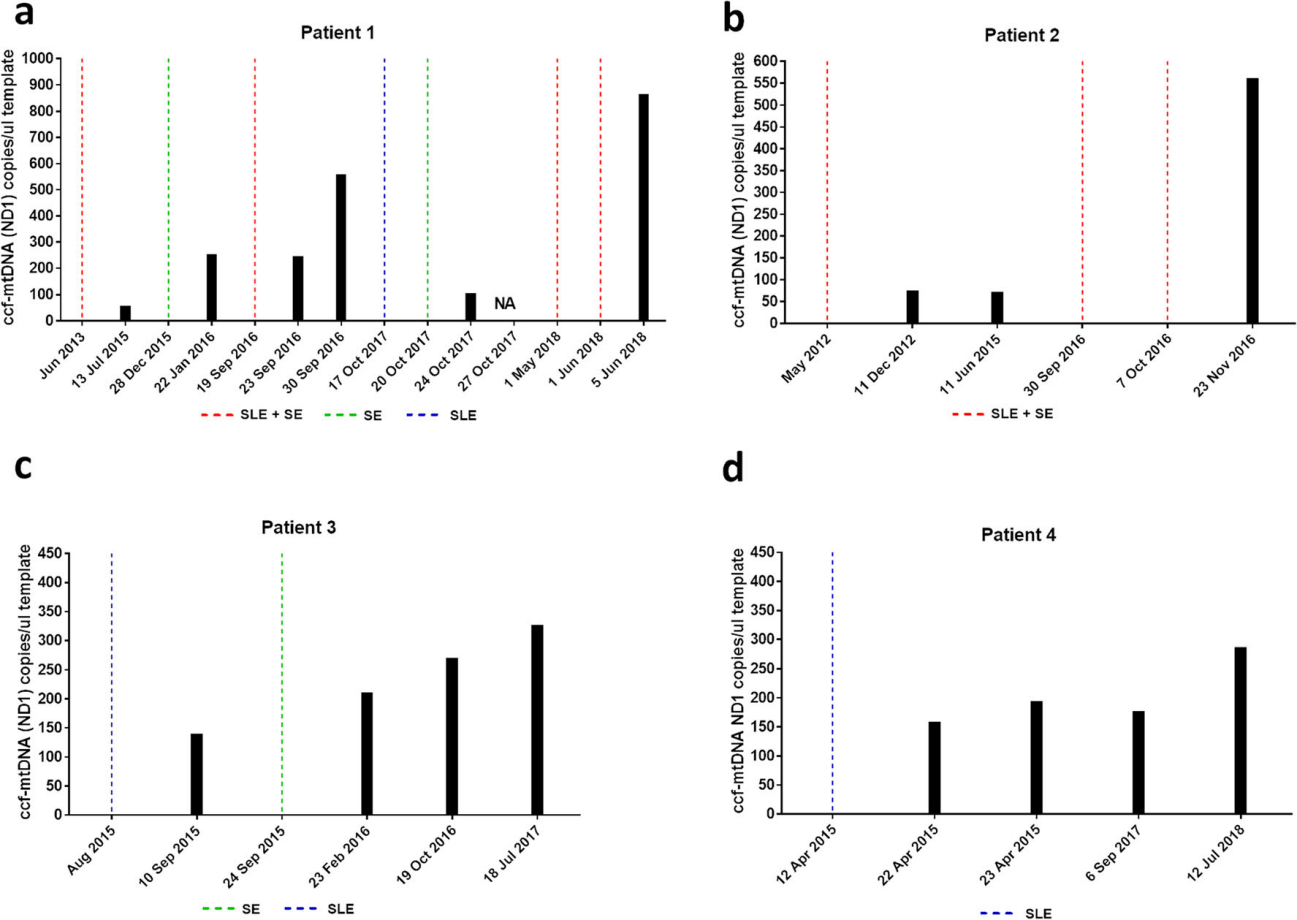
Evaluation of ccf-mtDNA in longitudinal samples of MELAS patients. **a-d** Ccf-mtDNA (*MT-ND2*) evaluated in longitudinal samples in four different patients. SLE, stroke-like episode; SE, status epilepticus

Furthermore, as we had the availability of longitudinal sampling in a few MELAS patients, we have been able to assess the ccf-mtDNA levels at multiple time-points in relation to SLEs or status epilepticus (SE) and during inter-critical periods. Noticeably, all samples sufficiently close to SLEs, combined or not with SE, clearly showed increased levels of ccf-mtDNA, which however were of different magnitudes in the different SLEs. This pattern was clearly observed in patients 1 and 2 (Fig. 2a–b, Supplementary Fig. 3a-b). In the other two patients, besides the increased ccf-mtDNA levels after the SLE or SE events, a pattern of progression over time was evident in a period of 2–3 years free of acute events (Fig. 2c–d, Supplementary Fig. 3c-d). All together, these results point to increase of ccf-mtDNA levels marking either the critical acute events or the progression of neurodegeneration in MELAS.

### Creatine elevation in serum is not associated to a specific clinical phenotype

Creatine was reported as the top hit in a study of global metabolic profiling in cell models of OXPHOS impairment and validated in human patients with miscellaneous mitochondrial disorders [19, 25, 26]. Thus, our next analysis was the assessment of creatine serum levels in our cohort, excluding patients who were taking creatine at the time of sample collection.

**Fig. 3:**
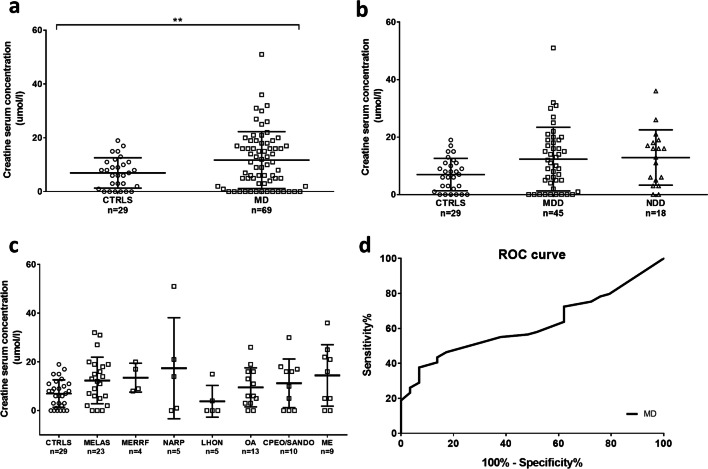
Evaluation of creatine in serum from controls and mitochondrial patients. **a** Creatine levels in controls (CTRLS) and mitochondrial patients (MD). **b** Creatine levels in CTRLS, patients with mtDNA genetic defects (MDD), and nuclear DNA genetic defects (NDD). **c** Creatine levels in CTRLS and mitochondrial patients stratified by phenotypes. **d** ROC curve for creatine in MD patients. AUC was 0.62 (95% CI 0.51–0.73, *p* = 0.06) for MD. **p value < 0.01

The overall comparison between controls and patients demonstrated a significant increase of creatine levels in the MD patient group (Table 1; Fig. 3a), which again was not influenced by gender and age (Supplementary Fig. 4a-b). However, comparing the creatine levels of CTRLS, MDD, and NDD, there were no significant differences among these three categories (Fig. 3b). Moreover, by stratifying patients into phenotypic categories, we did not find significant associations with any of the clinical phenotypes, and patients with creatine levels exceeding the control group range were similarly spread in all phenotypic groups, with the exception of MERRF and LHON (Fig. 3c). Considering the miscellaneous subgrouping of the cases above the control’s range, the large majority of these patients were affected by severe myopathy and/or neurological impairment.

Setting a cut-off threshold of 16 umol/l, which represented the 95th percentile of control values, creatine sensitivity and specificity for mitochondrial disease patients were 39% (95% CI 28–50%) and 93% (95% CI 84–102%), respectively. ROC curve was generated for the MD group (Fig. 3d), and the AUC was 0.62 (95% CI 0.51–0.73, *p* = 0.06). These results confirmed the previously reported low efficiency of creatine in discriminating mitochondrial patients from healthy individuals [25].

### FGF21 and GDF-15 significantly hallmark mitochondrial encephalomyopathy due to tRNA mutations (MELAS and MERRF)

Previous studies validated both FGF21 and GDF-15 as useful biomarkers in mitochondrial myopathies, even if not highly specific [17, 18, 27].

**Fig. 4:**
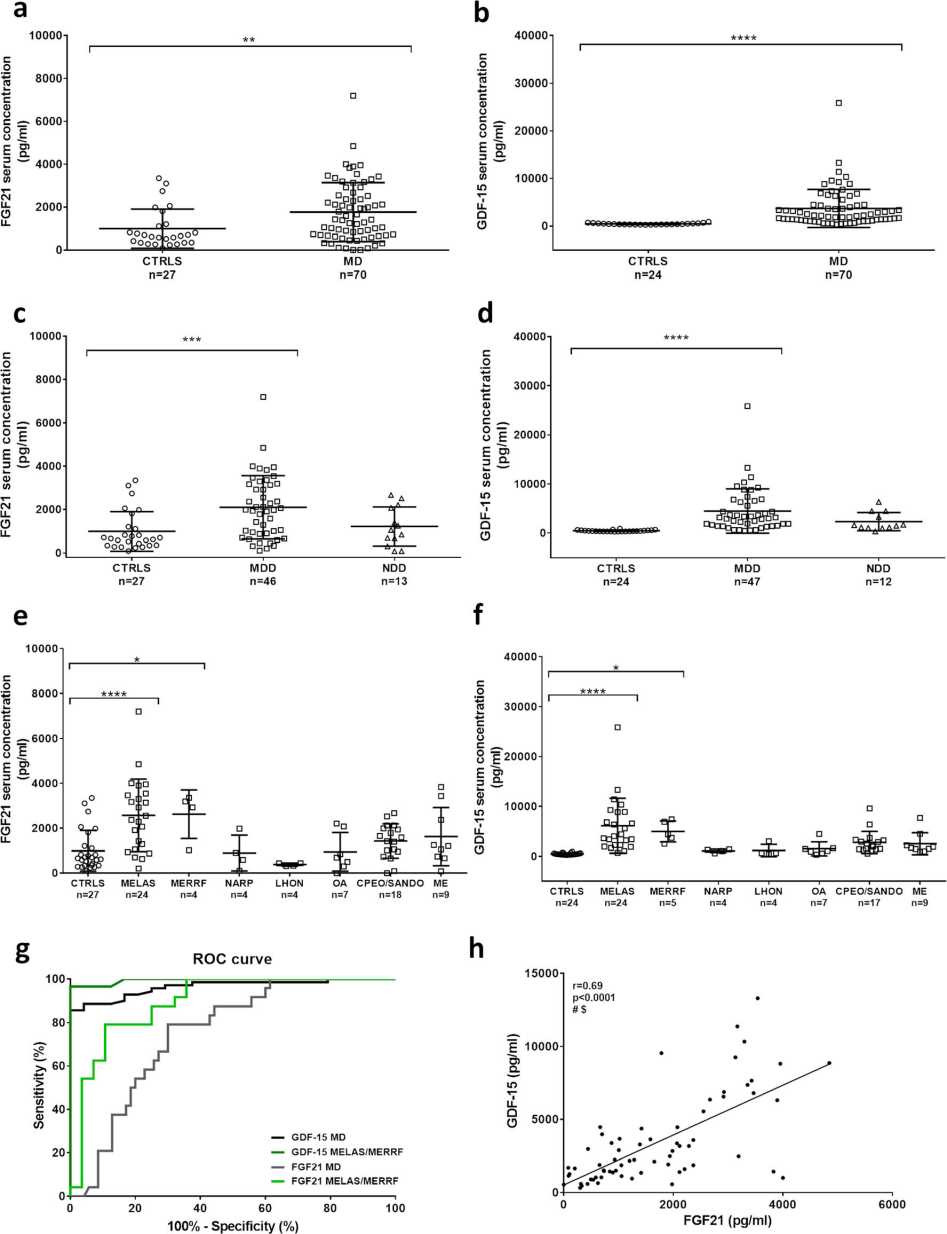
Assessment of FGF21 and GDF-15 in serum from controls and mitochondrial patients. **a**–**b** FGF21 and GDF-15 in controls (CTRLS) and mitochondrial patients (MD). **c**–**d** FGF21 and GDF-15 levels in CTRLS, patients with mtDNA genetic defects (MDD), and nuclear DNA genetic defects (NDD). **e**–**f** FGF21 and GDF-15 in CTRLS and mitochondrial patients stratified by phenotypes. **g** ROC curves for FGF21 and GDF-15 in MD and MELAS/MERRF patients. AUC for FGF21 was 0.75 (95% CI 0.65–0.85, *p* < 0.01) in MD and 0.89 (95% CI 0.80–0.98, *p* < 0.0001) in MELAS/MERRF, whereas AUC for GDF-15 was 0.96 (95% CI 0.93–0.99, *p* < 0.0001) in MD and 0.99 (95% CI 0.98–1.00, *p* < 0.0001) in MELAS/MERRF. **h** Pearson’s correlation between FGF21 and GDF-15 in MD. One outlier belonging to the MELAS group was excluded for the correlation analysis. Pearson *r* and *p* value are indicated in the graph. #, Significance after Bonferroni’s correction (*p* < 0.005); $, significance after Benjamini–Hochberg’s correction (FDR 0.10). ****p value < 0.0001, ***p value < 0.001, *p value < 0.05

Our results clearly demonstrated a significant increase of both biomarkers in serum of mitochondrial disease patients (Table 1; Fig. 4a–b), which was significantly contributed by the MDD group as compared with the NDD group (Fig. 4c–d). There was no significant gender-related difference (Supplementary Fig. 4c-d), whereas a significant association with age emerged for GDF-15 in the control group only (Supplementary Fig. 4e-f). Stratifying by phenotypes, MELAS and MERRF were both the only significant groups, and the CPEO/SANDO group had a not significant but similar tendency (Fig. 4e–f). All together, these two phenotypic subgroups, i.e. patients with tRNA point mutations (MELAS and MERRF), confirm that mtDNA translation defects are hallmarked by FGF21 and GDF-15.

FGF21 sensitivity and specificity for MD group were 41% (95% CI 30–53%) and 92% (95% CI 81–103%), with a cut-off of 1947 pg/ml, whereas, considering MELAS and MERRF as a single group, sensitivity was 64% (95% CI 47–82%). GDF-15 sensitivity and specificity for MD group were 89% (95% CI 81–96%) and 92% (95% CI 81–103%), whereas sensitivity for MELAS/MERRF group was 97% (95% CI 90–103%), all being calculated setting a cut-off of 711 pg/ml. ROC curves indicated that both FGF21 and GDF-15 were good biomarkers for MD and even more for tRNA mutation diseases, but GDF-15 had the best performance (Fig. 4g). In fact, AUC for FGF21 was 0.75 (95% CI 0.65–0.85, *p* < 0.01) in MD and 0.89 (95% CI 0.80–0.98, *p* < 0.0001) in MELAS/MERRF, whereas AUC for GDF-15 was 0.96 (95% CI 0.93–0.99, *p* < 0.0001) in MD and 0.99 (95% CI 0.98–1.00, *p* < 0.0001) in MELAS/MERRF.

Last, the only significant correlation that we found among the four biomarkers was between FGF21 and GDF-15 (Fig. 4h; Supplementary Fig. 5), as similarly reported by others [17, 18].

### Ccf-mtDNA, creatine, FGF21, and GDF-15 correlate with alanine and lactic acid in MELAS patients

**Fig. 5:**
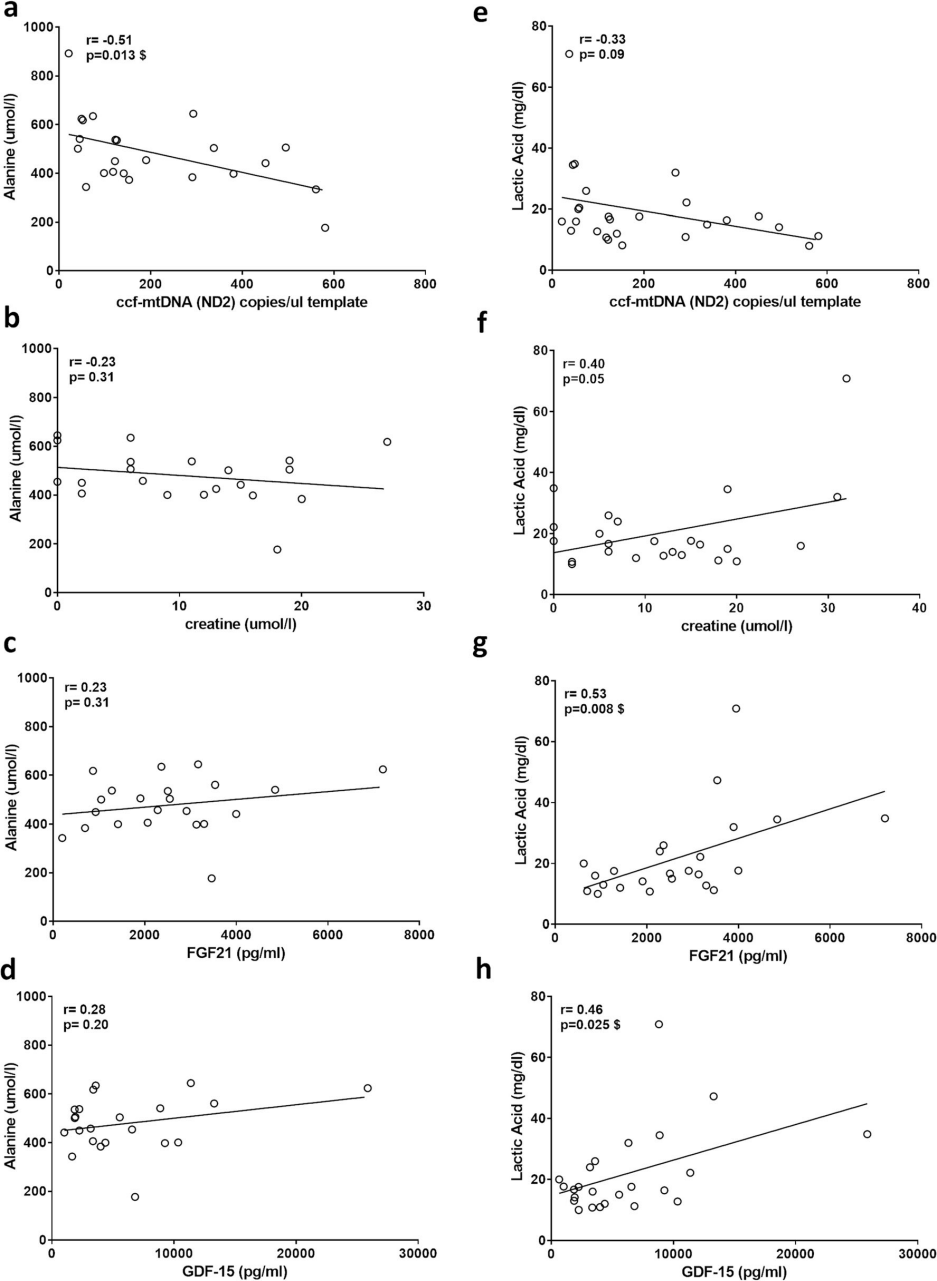
Correlation analyses between biomarkers and alanine/lactic acid plasma concentrations in MELAS patients. **a**–**d** Pearson’s correlation of alanine plasma concentration and ccf-mtDNA (*MT-ND2*), creatine, FGF21, and GDF-15. **e**–**h** Pearson’s correlation of lactic acid plasma concentration and ccf-mtDNA (*MT-ND2*), creatine, FGF21, and GDF-15. Pearson *r* and *p* values are indicated in the graphs. #, Significance after Bonferroni’s correction (*p* < 0.006); $, significance after Benjamini–Hochberg’s correction (FDR 0.10)

The MELAS subgroup was the most represented in our cohort of MD patients, and this is a condition defined by elevated lactic acidosis [1, 2, 4] for which, due to phenotypic severity, patients are strictly followed up. In fact, for MELAS patients, we had a consistent availability of plasma lactic acid and alanine measurements, which allowed for correlation analyses between the novel biomarkers assessed in the present study and these two classical biomarkers (Fig. 5). The only significant correlation for alanine was with ccf-mtDNA, but, interestingly, these two biomarkers negatively correlated, as shown by their inverse linear relationship (Fig. 5a). A similar tendency was observed also for lactic acid and ccf-mtDNA, although their association resulted weaker (Fig. 5e). Conversely, lactic acid positively correlated with creatine, FGF21, and GDF-15 (Fig. 5f–h).

## Discussion

Our study investigated a large cohort of patients with mixed primary mitochondrial pathology, mostly contributed by the epidemiological survey of the Emilia-Romagna Region in Italy (ER-MITO study), assessing multiple biomarkers, including the already validated FGF21, GDF-15, creatine, and, for the first time, the ccf-mtDNA. Significantly increased levels of all four biomarkers were found in the patients’ group, which, with the exclusion of creatine, we found to be most consistently contributed by the MDD subgroup. Our results on FGF21 and GDF-15 clearly confirmed, in an independent cohort, the previous findings, reproducing the striking association with mitochondrial translation defects due to tRNA mutations in MELAS and MERRF. Interestingly, only GDF-15 levels were correlated with age in controls. The ccf-mtDNA novel biomarker provided a strong association with MELAS. Remarkably, in the few cases of MELAS patients for which we had available longitudinal assessments, the increased levels of ccf-mtDNA matched the occurrence of SLEs or indicated the progression of neurodegeneration. Finally, yet in the MELAS group, we have been able to correlate the four biomarkers with the traditional hallmarks of OXPHOS dysfunction, namely the increase of lactic acid and alanine. A significant positive correlation was found between creatine, FGF21, GDF-15, and lactic acid, pointing to the contribution of skeletal muscle pathology to these biomarkers. Counterintuitively, we found a significant negative correlation of ccf-mtDNA with alanine (weaker for lactic acid) for which we hypothesize different timings in the plasma dynamic of these biomarkers in relation to the disease phase.

A few peculiar features differentiate mtDNA from the nuclear DNA. Among these, negligible CpG methylation, related to the ancestral bacterial origin of this small genome, makes mtDNA a potential immunogen able to induce immune and inflammatory responses when released outside mitochondria [28]. A seminal study by Zhang and colleagues in 2008 clearly demonstrated that traumatic injury and the related cell death enhance the extracellular release of mtDNA, as detected in plasma after injury, triggering inflammatory response [20]. In the following years, several studies evidenced increased levels of ccf-mtDNA in relation to different pathologic conditions, such as cancer, viral infection, or organ failure due to ischemic injury [21]. Contrary, low levels of ccf-mtDNA were found in the cerebral spinal fluid from patients affected by Alzheimer and Parkinson’s diseases, possibly explained by mtDNA depletion in neuronal cells [23, 29]. In our MELAS patients, the increased plasma levels of ccf-mtDNA may reflect the wave of neuronal loss occurring in concomitance with acute events, such as the SLEs, or the slower but consistent neuronal loss during the progression of neurodegeneration. This ccf-mtDNA increase possibly causes a state of still unrecognized inflammation in the central nervous system, which may be a relevant component of the pathological mechanism in MELAS, as indirectly suggested, for example, by some effectiveness of corticosteroids during SLEs [30, 31]. Clearly, additional studies are needed to clarify the origin and the role of this biomarker in MELAS and in other mitochondrial diseases characterized by acute/subacute development of neuronal loss (i.e. LHON and Leigh syndrome) and for which secondary inflammation may play a role in the cascade of pathogenic mechanisms and in the disease progression [32]. The timing of ccf-mtDNA increase should be also related, in the course of follow-up studies, with the other relevant biomarkers, including alanine and lactic acid. It is still unclear how SLEs may be accompanied by a systemic metabolic storm affecting peripheral tissues such as skeletal muscle, gastrointestinal neuromuscular component, and the major organs. Skeletal muscle, in particular, is believed to be the major source of plasma alanine and lactic acid, as well as myoglobin, creatine kinase, FGF21, and GDF-15, and the timing of their elevation may not be synchronous with SLEs, hallmarking different aspects of the pathological process. More in general, it might be worth investigating the potential of ccf-mtDNA in monitoring, by longitudinal follow-up assessments, natural history of mitochondrial diseases, and therapy response, including both therapies aimed at contrasting the disease etiology, as well as the potential benefit of anti-inflammatory therapies. In particular for MELAS, ccf-mtDNA represents a promising biomarker in evaluating the efficacy of recently proposed therapies aimed at preventing SLEs, such as arginine, citrulline, and taurine supplementation [33, 34].

Creatine, through its conversion into phosphocreatine by creatine kinase, has a direct role in the intracellular ATP storage, key to maintenance of the energy status of cells, and particularly important for muscle and nervous tissues [35]. Creatine has been previously proposed as a biomarker for mitochondrial diseases. In particular, increased plasma levels of this metabolite have been described for MELAS, MERRF, mtDNA depletion/deletions diseases, infantile-onset spinocerebellar ataxia (IOSCA), and respiratory chain deficiencies [19, 25, 26]. Although we showed significantly increased serum levels of creatine in our cohort of mitochondrial patients, we failed to find any association with a specific clinical phenotype. We emphasize that creatine was the only biomarker not able to differentiate MDD from NDD. The heterogeneous grouping of patients with elevated creatine levels shared a severe clinical condition, suggesting that increased blood-released creatine may reflect the levels of neurodegeneration or muscle impairment rather than being specific for a particular mitochondrial phenotype or genetic defect. We did not find any linear association between serum creatine and age. This result is apparently in contrast with previous studies reporting a decrease of plasmatic creatine from 12 to 20 years [25, 36], but the limited number of patients under 20 years in our cohort may explain the current findings (5/74). Importantly, creatine is often included in the pharmacological cocktails that are administered to mitochondrial patients, masking its informativeness in these cases. Thus, the use of creatine as biomarker for mitochondrial diseases has a few potential limitations, mainly the age-dependent variations and the confounder of its use as supplement in mitochondrial patients.

FGF21 and GDF-15 are cytokines with both autocrine and paracrine effects, released after induction of the integrated mitochondrial stress response, which in mammals is activated by defects in mtDNA expression (mtDNA maintenance and mitochondrial protein synthesis) [37–39]. Accordingly, several studies evidenced increased levels of these two factors in blood of mitochondrial patients, proposing them as useful biomarkers for these pathologies, especially for diseases due to defective mtDNA translation or maintenance [16–18, 40]. However, elevated FGF21 and GDF-15 in blood have been found also in non-mitochondrial myopathies and, more in general, in non-mitochondrial diseases [27, 36, 40]. Our results confirmed the association between FGF21/GDF-15 and mitochondrial diseases due to translation defects (MELAS and MERRF) with skeletal muscle involvement. Interestingly, in our results, these two biomarkers could not distinguish NDD patients, possibly due to the miscellaneous nature of this group and the limited number of patients compared with the MDD group. Lastly, in the control group, we found a significant association between GDF-15 and age, as reported by others [41], which potentially links this cytokine and aging. The positive correlation of FGF21/GDF-15 with the traditional biomarkers [36] just remarks the prevalent contribution of skeletal muscle OXPHOS impairment.

In conclusion, our study contributed the first exploratory results on the potential of a new biomarker for mitochondrial diseases, the ccf-mtDNA, which seems to hallmark phenotypes with very active neurodegeneration such as MELAS, and further confirmed the validity of FGF21 and GDF-15 as useful biomarkers, mostly sensing mitochondrial myopathy. The systematic use of these biomarkers in longitudinal follow-up studies will be crucial to define their significance in documenting disease progression and natural history, as well as their usefulness as surrogate biomarkers to clinical endpoints for therapeutic efficacy in trials.

## Electronic supplementary material

ESM 1 (DOCX 602 kb)

ESM 2 (DOCX 17 kb)
