# Supplementary material for: Expanding and validating the biomarkers for mitochondrial diseases
Source: J Mol Med (Berl). 2020 Aug 26;98(10):1467–78. doi: 10.1007/s00109-020-01967-y (PMC7524861; doi:10.1007/s00109-020-01967-y)
Supplement: Supplementary file 1 — (DOCX 602 kb) [file 109_2020_1967_MOESM1_ESM.docx]

**Electronic Supplementary material**

**Journal of Molecular Medicine**

**“Expanding and validating the biomarkers for mitochondrial diseases”**

Alessandra Maresca, PhD^1^, Valentina Del Dotto, PhD^2^, Martina Romagnoli, PhD^1^, Chiara La Morgia, MD, PhD ^1,2^, Lidia Di Vito, MD^1^, Mariantonietta Capristo, PhD^1^, Maria Lucia Valentino^1,2^, MD, Valerio Carelli, MD, PhD, ^1,2*^ and the ER-MITO study group.

^1^IRCCS Istituto delle Scienze Neurologiche di Bologna, UOC Clinica Neurologica, Bologna, Italy

^2^Department of Biomedical and Neuromotor Sciences, University of Bologna, Bologna, Italy

^*^Corresponding author:

Valerio Carelli, MD, PhD

Department of Biomedical and Neuromotor Sciences, University of Bologna

IRCCS Istituto delle Scienze Neurologiche di Bologna

via Altura 3, 40139, Bologna, Italy

e-mail: [valerio.carelli@unibo.it](mailto:valerio.carelli@unibo.it)

**List of content:**

Supplementary Table 1

Supplementary Fig. 1

Supplementary Fig. 2

Supplementary Fig. 3

Supplementary Fig. 4

Supplementary Fig. 5

**Supplementary Table 1- Mitochondrial patients categorized into phenotypic classes.**

| **Phenotype** | **Genome** | **Gene** | **Males** | **Females** | **No.** |
| --- | --- | --- | --- | --- | --- |
| **MELAS**  **(28)** | mtDNA | *MT-TL1* | 16 | 11 | 27 |
|  |  | *MT-ND3* | - | 1 | 1 |
| **MERRF**  **(6)** | mtDNA | *MT-TK* | 3 | 3 | 6 |
| **NARP**  **(6)** | mtDNA | *MT-ATP6* | 4 | 2 | 6 |
| **LHON**  **(34)** | mtDNA | *MT-ND1* | 6 | 1 | 7 |
|  |  | *MT-ND4* | 17 | 5 | 22 |
|  |  | *MT-ND6* | 3 | - | 3 |
|  | *unknown* |  | 1 | 1 | 2 |
| **Optic Atrophy**  **(16)** | nDNA | *OPA1* | 3 | 3 | 6 |
|  |  | *SLC25A46* | 1 | - | 1 |
|  |  | *SDHA* | 1 | - | 1 |
|  |  | *IBA57* | 1 | - | 1 |
|  |  | *SSBP1* | 2 | - | 2 |
|  | *unknown* |  | 3 | 2 | 5 |
| **CPEO/SANDO**  **(21)** | mtDNA | *Single deletion* | - | 4 | 4 |
|  |  | *MT-TY* | 1 | - | 1 |
|  |  | *MT-TL1* | 2 | - | 2 |
|  |  | *MT-CO2* | 1 | - | 1 |
|  | nDNA | *OPA1/TWNK* | 1 | - | 1 |
|  |  | *TWNK* | - | 1 | 1 |
|  |  | *POLG* | 1 | 3 | 4 |
|  |  | *DNA2* | - | 1 | 1 |
|  | *unknown* |  | - | 6 | 6 |
| **Mitochondrial Encephalo-myopathy (12)** | mtDNA | *Single deletion* | 1 | 1 | 2 |
|  |  | *MT-TM* | 1 | - | 1 |
|  |  | *MT-ND3* | 1 | - | 1 |
|  |  | *MT-CO2* | 1 | - | 1 |
|  | nDNA | *MGME1* | 1 | - | 1 |
|  |  | *SUCLA2* | 1 | - | 1 |
|  |  | *SFNX4* | - | 1 | 1 |
|  |  | *RARS2* | 1 | - | 1 |
|  | *unknown* |  | 2 | 1 | 3 |

Abbreviations: MT-TL1, transfer RNA Leucine; MT-TK, transfer RNA Lysine; MT-TY, transfer RNA Tyrosine; MT-TM, transfer RNA Methionine; MT-ND3, NADH dehydrogenase 3; MT-ATP6, ATP synthase 6; MT-ND1, NADH dehydrogenase 1; MT-ND4, NADH dehydrogenase 4; MT-ND6, NADH dehydrogenase 6; MT-CO3, cytochrome c oxidase III; MT-CO2, cytochrome c oxidase II; OPA1, Optic Atrophy 1; SLC25A46, Solute Carrier Family 25 Member 46; SDHA, Succinate Dehydrogenase Complex Flavoprotein Subunit A; IBA57, Iron-Sulfur Cluster Assembly Factor; SSBP1, Single strand binding protein 1; TWNK, Twinkle MtDNA Helicase; POLG, DNA Polymerase Gamma, Catalytic Subunit; DNA2, DNA replication ATP-dependent helicase/nuclease DNA2; MGME1, Mitochondrial Genome Maintenance Exonuclease 1; SUCLA2, Succinate-CoA Ligase ADP-Forming Subunit Beta; SFXN4, Sideroflexin-4; RARS2, Arginyl-TRNA Synthetase 2, Mitochondrial.

**Supplementary Figure 1**

**
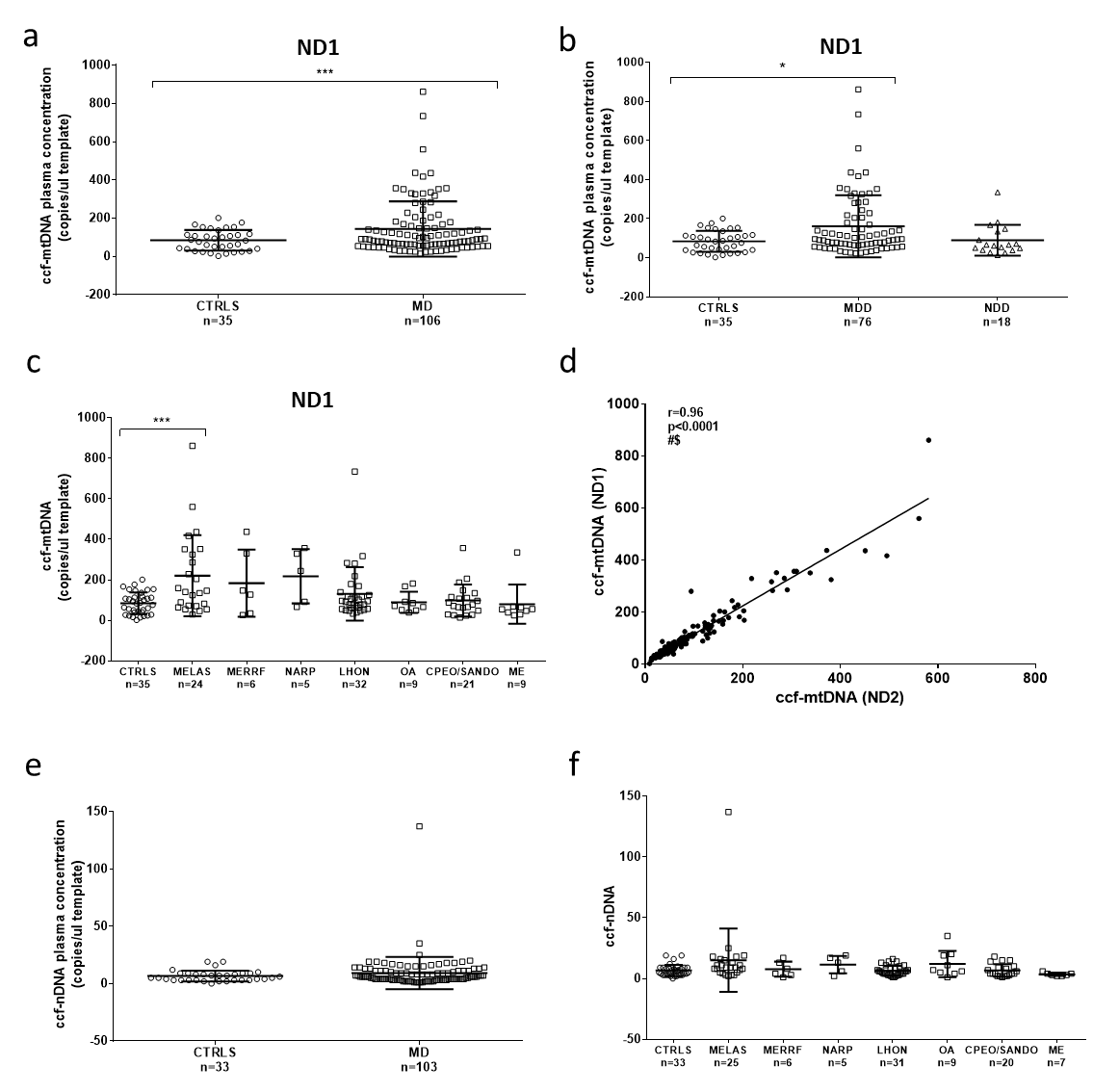
**

**Supplementary Fig. 1** Ccf- mtDNA and ccf-nDNA evaluation in plasma from controls and mitochondrial patients.

**(a)** Ccf-mtDNA (*MT-ND1*) in controls (CTRLS) and mitochondrial patients (MD). **(b)** Ccf-mtDNA (*MT-ND1*) in CTRLS, patients with mtDNA genetic defects (MDD) and nuclear DNA genetic defects (NDD) **(c)** Ccf-mtDNA (*MT-ND1*) in CTRLS and mitochondrial patients stratified by phenotypes. **(d)** Pearson’s correlation between *MT-ND2* and *MT-ND1* assays for ccf-mtDNA. # Significance after Bonferroni’s correction (p<0.005), $ significance after Benjamini–Hochberg correction (FDR 0.10). **(e)** Ccf-nDNA in CTRLS and MD patients. **(f)** Ccf-nDNA in CTRLS and mitochondrial patients stratified by phenotypes. * p-value <0.05, *** p-value < 0.001.

**
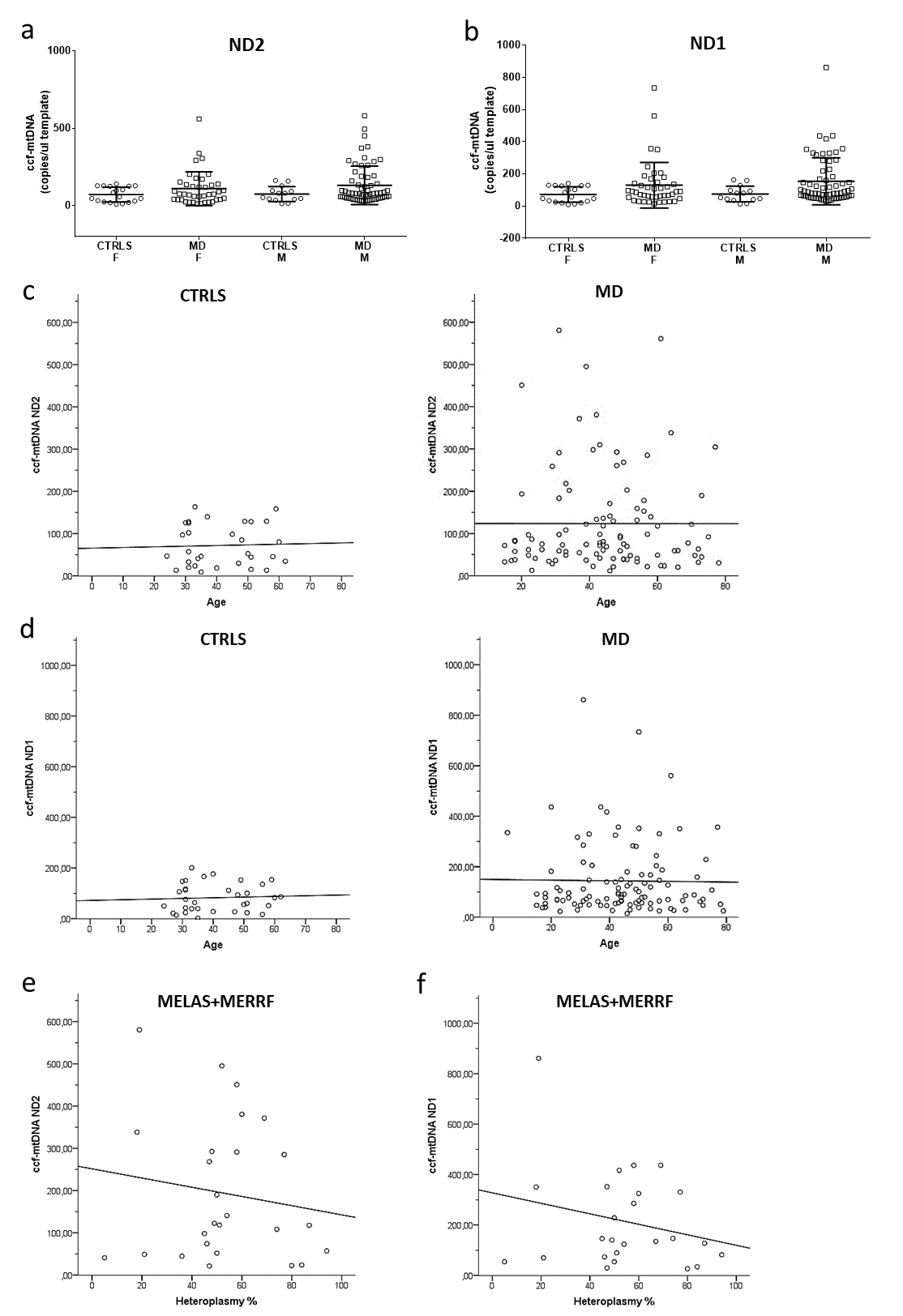
Supplementary Figure 2**

**Supplementary Fig. 2** Sex, age and heteroplasmy influence on ccf-mtDNA.

**(a-b)** Ccf-mtDNA shown for females and males in CTRLS and MD patients. **(c-d)** Linear regression for ccf-mtDNA and age in CTRLS and MD. No significant association was found. **(e-f)** Pearson’s correlation between ccf-mtDNA and heteroplasmy levels of m.3243 and m.8344 mutations in MELAS and MERRF merged group. No significant correlation was found.


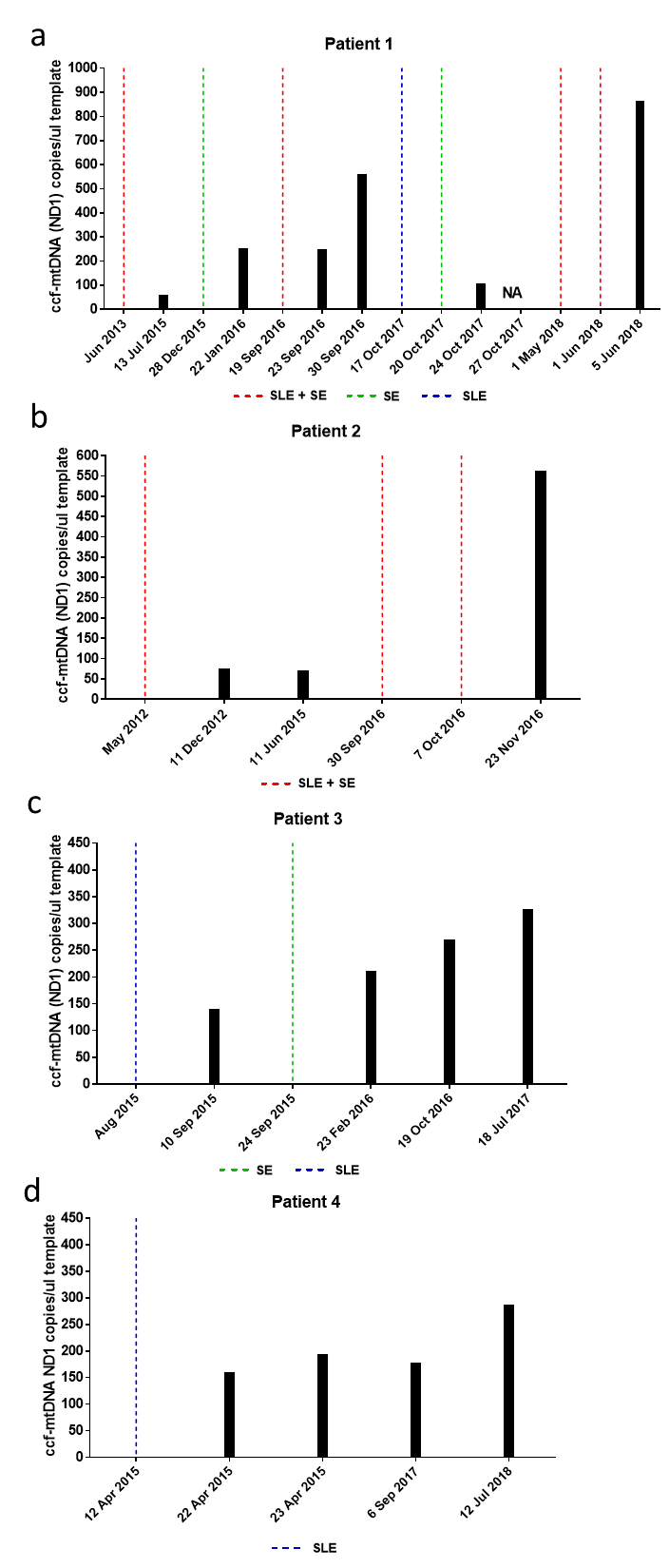
**Supplementary Figure 3**

**Supplementary Fig. 3** Evaluation of ccf-mtDNA in longitudinal samples of MELAS patients.

Ccf-mtDNA (*MT-ND1*) evaluated in longitudinal samples in four different patients. SLE: stroke-like episode, SE: status epilecticus.

**
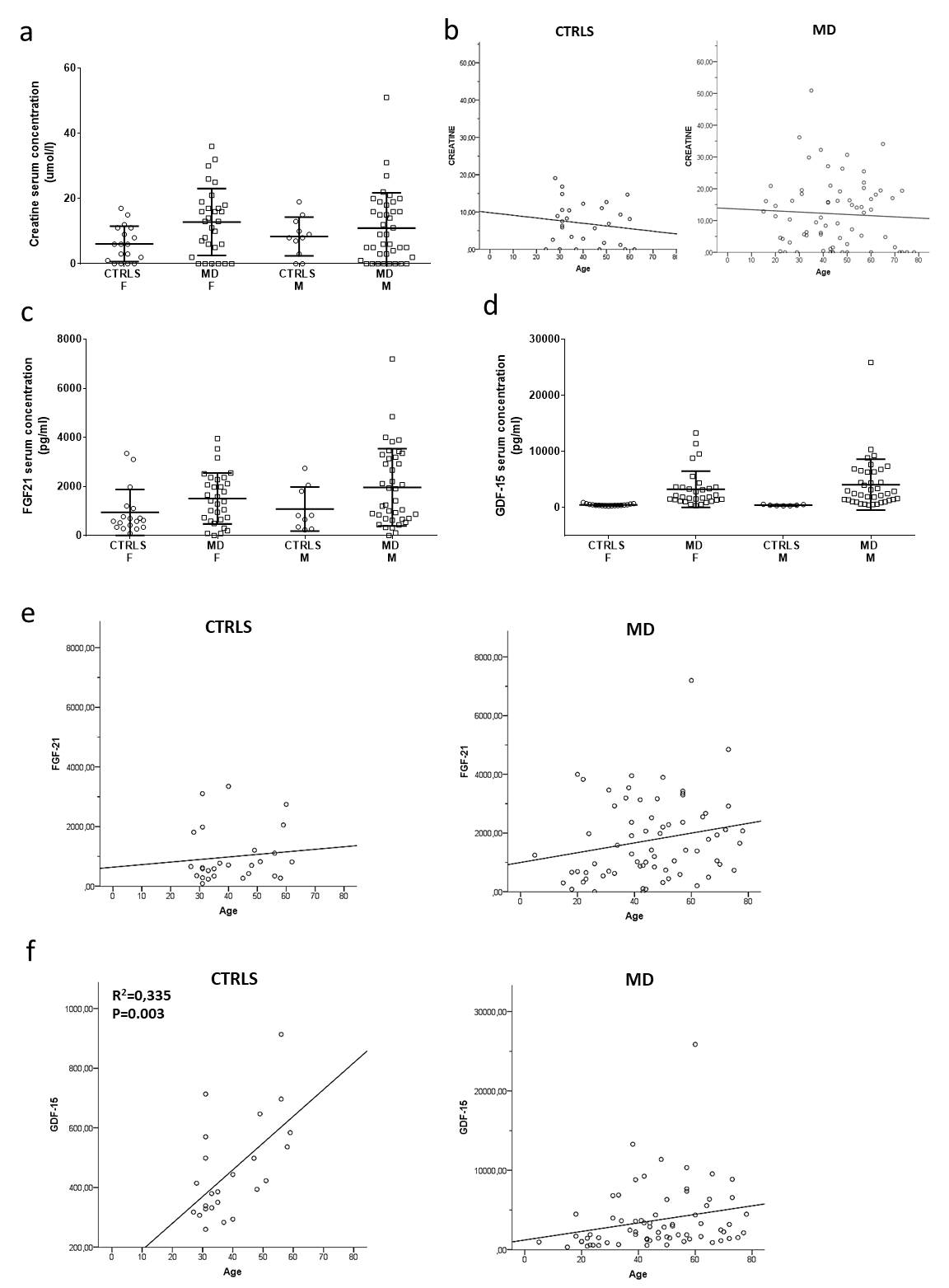
Supplementary Figure 4**

**Supplementary Fig. 4** Gender stratification and linear regression with age for creatine, FGF21 and GDF-15.

**(a)** Creatine shown for females and males in CTRLS and MD. **(b)** Linear regression for creatine and age in CTRLS and MD. No significant association was found. **(c-d)** FGF21 and GDF-15 shown for females and males in CTRLS and MD. **(e-f)** Linear regression for FGF21 **(e)** and GDF-15 **(f)** and age in CTRLS and MD. A significant association was found for GDF-15 and age in CTRLS R^2^ = 0.335, p-value = 0.003.

**Supplementary Figure 5**

**Supplementary Fig. 5** Correlation analyses amongst ccf-mtDNA, creatine, FGF21 and GDF-15.

Pearson’s correlation analyses amongst the four biomarkers of this study. The analysis was performed for both ccf-mDNA assays (*MT-ND2* and *MT-ND1*). Graphs of Pearson’s correlations for FGF21/GDF-15 and *MT-ND2*/*MT-ND1* are reported in Fig. 4h and in Supplementary Fig. 1d, respectively. NS Non significant after Bonferroni’s and Benjamini–Hochberg’s corrections (FDR 0.10).
