# Supplementary material for: Expanding and validating the biomarkers for mitochondrial diseases
Source: J Mol Med (Berl). 2020 Aug 26;98(10):1467–78. doi: 10.1007/s00109-020-01967-y (PMC7524861; doi:10.1007/s00109-020-01967-y)
Supplement: Supplementary file 2 — (DOCX 17 kb) [file 109_2020_1967_MOESM2_ESM.docx]

**ER-MITO Study Group participants**

Patrizia Avoni^1,2^, Rocco Liguori^1,2^, Vitantonio Di Stasi^1^, Paolo Tinuper^1,2^, Michele Carbonelli^1^, Leonardo Caporali^1^, Francesca Tagliavini^1^, Flavia Palombo^1^,

Antonella Pini^3^, Melania Giannotta^3^, Giuseppe Gobbi^3^, Duccio Maria Cordelli^4^, Emilia Ricci^4^, Emilio Franzoni^4^, Gaetano Procaccianti^5^, Mauro Gentile^5^, Andrea Zini^5^, Maria Guarino^6^, Rita Rinaldi^6^, Fabio Cirignotta^6^,

Giacomo Biasucci^7^, Donata Guidetti^8^, Paola De Mitri^8^, Eugenia Rota^8^, Paolo Immovilli^8^,

Francesco Pisani^9^, Emanuela Claudia Turco^9^, Benedetta Piccolo^9^, Vladimiro Pietrini^10^, Umberto Scoditti^10^, Lilia Latte^10^, Paola Castellini^10^, Arens Taga^10^, Laura Delaj^10^, Maria Federica Bellanova^11^ Enrico Montanari^12^, Graziella Pedà^12^, Letizia Manneschi^13^, Tullia Ferrante^13^,

Elvio Della Giustina^14^, Alessandro Iodice^14^, Carlotta Spagnoli^14^, Carlo Fusco^14^, Ylenia Maini^14^, Norina Marcello^15^, Marialuisa Zedde^15^,

Patrizia Bergonzini^16^, Elisa Caramaschi^16^, Azzurra Guerra^16^, Alessandra Ariatti^17^, Guido Bigliardi^17^, Maria Luisa Dell'Acqua^17^, Stefano Meletti^17,18^, Paolo Nichelli^17,18^, Laura Vandelli^17^, Patrizia Sola^17^, Mario Santangelo^19^,

Raffaella Faggioli^20^, Luisa Maria Caniatti^21^, Valeria Tugnoli^21^, Cristiano Azzini^21^, Enrico Granieri^21^,

Gabriele Ciucci^22^, Pietro Querzani^22^, Claudio Callegarini^22^, Ricciardelli Paolo^23^,

Giustino Melideo^24^, Giovanni Tricomi^24^, Marcello Stella^25^, Walter Neri^26^, Carlo Guidi^26^, Giampiero Galletti ^26^, Yasmin Handouk^27^, Maria Grazia Passarin^27^,

Mariarosaria Gullì^28^, Sarajlija Jasenka^28^, Alessandro Ravasio^29^, Beatrice Viti^29^, Marco Curro Dossi^29^,

Vittoria Mussuto^30^

Roberto D’Alessandro^31^, Cristina Fonti^31^, Corrado Zanesini^31^, Elisa Baldin^31^.

^1^IRCCS Istituto delle Scienze Neurologiche di Bologna, UOC Clinica Neurologica, Bologna, Italy.

^2^Dipartimento di Scienze Biomediche e Neuromotorie, Università di Bologna, Bologna, Italy.

^3^IRCCS Istituto delle Scienze Neurologiche di Bologna, UOC Neuropsichiatria Infantile, Bologna, Italy.

^4^U.O. Neuropsichiatria infantile, Policlinico S.Orsola-Malpighi, Università di Bologna, Bologna, Italy.

^5^IRCCS Istituto delle Scienze Neurologiche di Bologna, UOC Neurologia e Rete Stroke Metropolitana, Ospedale Maggiore, Bologna, Italy.

^6^IRCCS Istituto delle Scienze Neurologiche di Bologna, UOC interaziendale Clinica Neurologica Metropolitana (NeuroMet), Bologna, Italy.

^7^UO Pediatria e Neonatologia, Ospedale Guglielmo da Saliceto Piacenza, Italy.

^8^UO Neurologia, Ospedale Guglielmo da Saliceto, Piacenza, Italy.

^9^Unità di Neuropsichiatria Infantile, Ospedale Maggiore, AOU di Parma, Italy.

^10^UO di Neurologia, AOU di Parma, Italy.

^11^UO di Neurologia, Lab. di Neuropatologia, AOU di Parma, Italy.

^12^UO Neurologia, Ospedale di Vaio-Fidenza, Italy.

^13^UO di Neurologia, P. O. di Fidenza San Secondo AUSL di Parma, Italy.

^14^Unita' di Neuropsichiatria Infantile Ospedale S. Maria Nuova Reggio Emilia, Italy.

^15^SOC Neurologia, Azienda Unità Sanitaria Locale-IRCCS di Reggio Emilia Italy.

^16^Neurologia Pediatrica, Clinica Pediatrica AOU di Modena, Italy.

^17^UO di Neurologia, Ospedale di Baggiovara, Modena, Italy.

^18^Dipartimento di Scienze Biomediche, Metaboliche e Neuroscienze, Università di Modena e Reggio Emilia, Italy.

^19^UO di Neurologia, Ospedale Ramazzini, Carpi, Modena, Italy.

^20^Sezione di Neurologia Pediatrica, Clinica Pediatrica, Ospedale S. Anna, AOU di Ferrara, Italy.

^21^UO di Neurologia, Ospedale S. Anna, AOU di Ferrara, Italy.

^22^UOC Neurologia Ravenna, AUSL della Romagna, Italy.

^23^Servizio di Neurologia Pediatrica, Ospedale di Faenza, AUSL di Ravenna, Italy.

^24^U.O. Neuropsichiatria della Infanzia e della Adolescenza – Azienda Unità Sanitaria Locale della Romagna, Cesena, Italy.

^25^Pediatria e Terapia Intensiva Neonatale-Pediatrica - Ospedale di Cesena, Italy.

^26^UO di Neurologia, AUSL di Forlì, Italy.

^27^UO di Neurologia, Ospedale Bufalini Cesena, Italy.

^28^Neuropsichiatria, Ospedale di Rimini, Italy.

^29^Servizio di Neurologia, Ospedale di Rimini, Italy.

^30^UO di Neurologia, AUSL Imola, Italy.

^31^IRCCS Istituto delle Scienze Neurologiche di Bologna, UO Epidemiologia e Statistica, Bologna, Italy.
